# Thermodynamic Evaluation of Electrode Storage for Capacitive Deionization

**DOI:** 10.1021/acsomega.4c08707

**Published:** 2025-03-04

**Authors:** Daniel Moreno, Hunter Nelson, Grant Cary, Devon Parker, Pablo Skaggs

**Affiliations:** Missouri State University, Springfield, Missouri 65897, United States

## Abstract

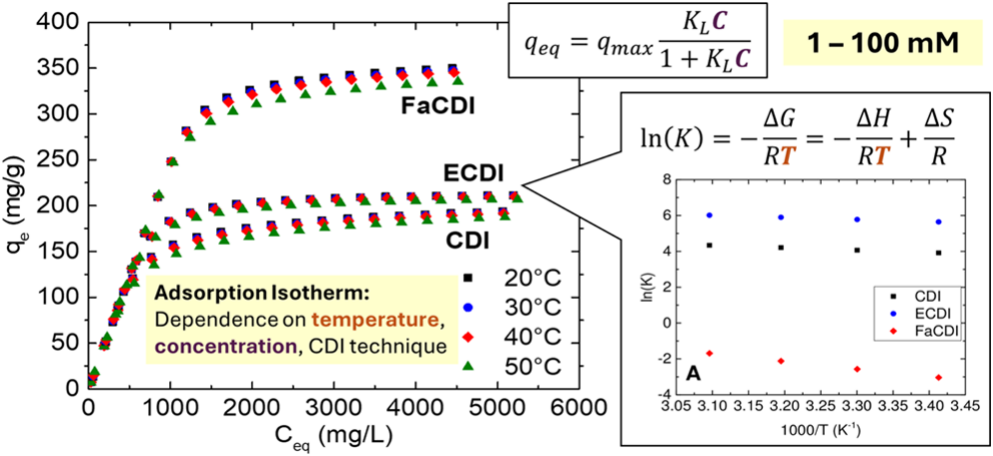

This study details the development of a computational adsorption model for predicting thermodynamic adsorption parameters for capacitive deionization (CDI) processes. To do this, multiple starting concentrations and temperatures are needed to predict the best fit value. This is first demonstrated experimentally using an in-house CDI cell with custom heaters, and determining maximum adsorption capabilities for a selected range of conditions. This has been done previously for CDI in the published literature, but here, experimental results are incorporated to provide the best fit to a computational model, which runs transient CDI tests in batch mode over multiple concentrations and temperatures to determine adsorption parameters. This saves the eventual challenge of having to run many different experiments independently to determine such adsorption parameters, the accuracy of which may be questionable subject to different experimental errors. With the model, many parameters can be quickly scanned at once, and adsorption parameters can be determined based on the concentration and temperature values selected, as well as other operating conditions, such as voltage and cell resistance. The computational isotherms are generated using the Gouy–Chapman–Stern (GCS) model, which is common for the lower concentration values used for CDI. The model also considers fixed and mobile chemical charges for enhanced CDI (ECDI) and Faradaic CDI (FaCDI), respectively, which have been examined as alternatives to improve CDI performance. While primarily proof-of-concept, the results obtained here demonstrate the benefits in adsorption capabilities, and energy savings obtained here demonstrate benefits in adsorption capabilities and energy savings for FaCDI, coinciding with higher enthalpies and entropies of adsorption. The model also serves as a benchmark in the future for how the results can be further explored and better fits can be obtained experimentally to confirm stability in the thermodynamic values.

## Introduction

Global demand for clean water continues to increase, and up to half of the world’s population could be living in areas facing water scarcity as early as the year 2025.^1^ With clean water constituting a small portion of the planet’s available water, the need for clean and efficient water purification processes are only going to become more necessary in the future. State-of-the-art purification methods include pressure-based (reverse osmosis, or RO), or thermal-based distillation processes.^2^ For RO, the pressures required can be substantial; generally around 2–17 bar for brackish water and in the range of 40–82 bar for seawater.^3,4^ The seawater also has an osmotic pressure of approximately 27 bar that must also be overcome. Energy requirements for seawater desalination via RO are in the range of 4–6 kWh/m^3^,^5^ though this can be as low as 1 kWh/m^3^ for brackish water,^6^ and values even below this threshold have been reached.^7^ Distillation is performed through a process called multistage flash where water is pushed to a higher pressure, and temperature is increased to separate freshwater from impurities.^8^ While distillation boasts advantages in a lower brine footprint to the environment and lower pressures required, energy requirements are generally higher than for RO, requiring values in the range of 55–80 kWh/m^3^,^9^ most of which is needed in the form of heat energy.^10^

This leaves a desire for a low-energy, cost-effective form of water purification that can be used to rival these current modern forms of desalination. Capacitive deionization (CDI) is one such technique that is cost-effective and energy efficiency, and can serve as an effective alternative to RO and MSF without the need for high temperatures or pressures to operate.^11^ CDI uses carbon-based porous electrodes with applied voltage to remove salt ions from saline solutions.^12^ This method is more effective when lower concentrations of salt (<100 mM) are used.

The operating principle of capacitive deionization (CDI) involves electrosorption of aqueous ions onto porous electrodes, effectively desalinating either a continuous or a batch feed stream.^13^ Porous electrodes are by far the most investigated CDI electrodes for charge storage owing to their high specific surface area, along with their good chemical and thermal stability. Among the materials used to construct these carbon electrodes include activated carbon fibers, aerogels, mesoporous, and carbon nanotubes.^14−17^ The electrosorption process is governed by the formation of an electrical double layer at the electrodes. Double layer theory is commonly described by using one of two models: either the Gouy–Chapman–Stern (GCS) model, or the more recently developed modified Donnan (mD) model, which considers overlapping double layers in regions with sufficiently small pore sizes.^18^ The mD model is typically employed at lower concentrations, *C* < 50 mM, which is of greater interest for CDI studies.

One recent technique is implementing chemical charges on surfaces of the electrodes,^19^ such as sulfonic, amine, or carboxylic groups on the micropores of the electrode materials.^20−22^ If chemical charges are introduced at the electrodes with the same sign of surface charge (i.e., opposite cell terminal), cell performance can be enhanced (enhanced CDI (ECDI)). However, this chemical charge can be further modified by using redox-active materials,^23^ such as surface-modified with redox-active polymers. By incorporating such redox-active species, chemical charges are not fixed but vary based on cell voltage and other related operating conditions. This allows for an effective tuning of the CDI process to enable an even more optimized performance. As the redox-active species promote Faradaic reactions but maintain traditional capacitor-like behavior, the charges were initially termed pseudocapacitive separations.^23^ However, some pseudocapacitors typically involve a separate mechanism through which ions are stored; the process has been more recently called Faradaic CDI (FaCDI), a terminology used in this study. Intercalation-based materials, such as manganese oxide (MnO_2_), and ruthenium oxide (RuO_2_), cannot be modeled similarly to a FaCDI process, since the redox reactions do not take place on the surface or the electrode, but rather with the porous electrode structure, thus requiring a full one-dimensional (1-D) analysis of charge storage inside the electrode.^24−26^ Previously, the performance of CDI, ECDI, and FaCDI had been developed and compared in short-term continuous mode desalination cycles; these studies also examined and compared ion concentration and point of zero charge (PZC).^27^

As electrodes can only hold a finite surface area, dictated by the macropores and micropores within the structure, there will be a limit to the maximum amount of ion removal that an electrode can achieve. By performing desalination tests over various concentrations and temperatures for a particular electrode to determine its storage limit, we can obtain thermodynamic adsorption parameters for the electrode can be obtained. Gibbs energy of adsorption–describes the spontaneity of the reaction, entropy characterizes the affinity (randomness) of the sorbent/sorbate pair, and enthalpy characterizes the type of adsorption (physisorption or chemisorption) associated with the electrode.^28^ Experimentally, obtaining these parameters requires developing a cell capable of applying variable temperatures and examining the different types of electrode materials used. As this would require many experiments for different concentrations, temperatures, and electrode materials, there is value in first studying electrosorption characteristics computationally.

One such approach for evaluating electrosorption techniques for CDI is through density functional theory (DFT). DFT has been used in previous studies for CDI,^29,30^ and in predicting thermodynamic adsorption thermodynamics characteristics of electrodes.^31^ While electrochemical capacitors have been developed and established using DFT,^32,33^ limitations are noted in that only a portion of the electrode’s surface can be captured, and the models can often come with high computational costs.^34,35^ Nevertheless, it can prove useful in augmenting other computational and experimental results obtained such as the ones evaluated in this study.

Previously published experimental work displays only limited data for a small number of temperatures and concentration ranges, and the variation of the electrosorption characteristics estimated with the data points selected could be significant. Understanding the adsorption characteristics is useful to predict favorable sites on select material surfaces,^36^ improve upon said modifications effectively,^37^ and apply appropriate modifications to the site to improve performance via these characteristics.^38^

In this article, a thermodynamic isotherm is used to obtain enthalpy/entropy of adsorption values. Experiments will be run at varying concentrations and temperatures using an in-house setup for CDI using carbon-based electrodes. This enables the prediction of some thermodynamic adsorption values for enthalpy and enthalpy based on purely experimental work. The results were then compared with the developed computational model to determine the appropriate parameters to use for establishing the best fit. From there, the model can be expanded to a range of different parameters such as voltage, internal/external resistance, and other material properties (estimated from experimental results) and determine their effect on the enthalpy and entropy of adsorption. The model will also display the effects of ECDI and FaCDI, adding a fixed or variable chemical charge to alter the electrochemical performance. Here, it is shown not only that energy efficiency is higher when the FaCDI method of removal is used but also that salt removal is maximized under the same set of conditions and can produce greater enthalpy/entropy of adsorption values when the removal is highest.

The CDI, ECDI, and FaCDI processes will all be compared by looking at equilibrium conditions for ideal, fully reversible cycles. Second, computational performance will be examined in short-term batch mode simulations to examine adsorption isotherms and compare standard values of adsorption. By examining these terms of adsorption, spontaneity, randomness, and whether the process is exothermic or endothermic can be determined. As computational studies are not typically done for evaluating such characteristics of adsorption processes, the results obtained and discussed can be of future benefit for studying a wide range of different adsorption processes. They can also provide a useful starting point for more involved computational studies, such as DFT or molecular dynamics (MD), to be developed and compared.

## Experimental Section

To begin the experimental procedure, the electrode material in the flow cell must be created. The components needed for the construction of the electrode material are a variable temperature furnace, scale, graphite foil, aluminum foil, activated charcoal, mesoporous carbon black, Poly(tetrafluoroethylene) (PTFE) binder, ethanol, beaker, hot plate, and a hand roller. The composition of the activated charcoal, carbon black, and binder will be 0.850, 0.05, and 1675 g (respectively). These respective amounts combined in a beaker with roughly 10 mL of ethanol will be placed on the hot plate at a temperature of 130 °C and a stirring rate of roughly 200 rpm. This will occur until the ethanol boils off to create a dough-like consistency paste of the electrode material. This paste will be placed on a graphite foil sheet roughly 3 × 4 in.^2^ covered in foil and rolled by hand to spread the electrode material uniformly across the foil. The completed electrode will then be placed in the furnace at a temperature of 150 °C for a time of 3 h. This furnace temperature and time were motivated by a series of preliminary tests evaluating the effects on the preparation time of the electrode on the overall salt removal capabilities (Table S1).

The surface area per gram can be estimated based on the characteristics of the carbon black primary used to make the electrode. Surface areas used for carbon-based electrodes for CDI are typically on the order of 1000 m^2^/g.^39,40^ Pore size distribution, not measured in this study, but estimated as 1–3 nm.^41^ This is typically the threshold between macropores and micropores. Pore sizes are predicted using gas adsorption isotherms, different from the ones reported in this study. Generally, in the modified Donnan theory, the pores are in the 2–4 nm range,^18^ corresponding with this average. Electrical conductivity for CDI electrodes is generally high, on the order of 1–100 S/cm.^42,43^ Thus, its contribution to the electrical resistance of the cell can generally be neglected, relative to the ionic conductivity in the electrode.

**Figure 1:**
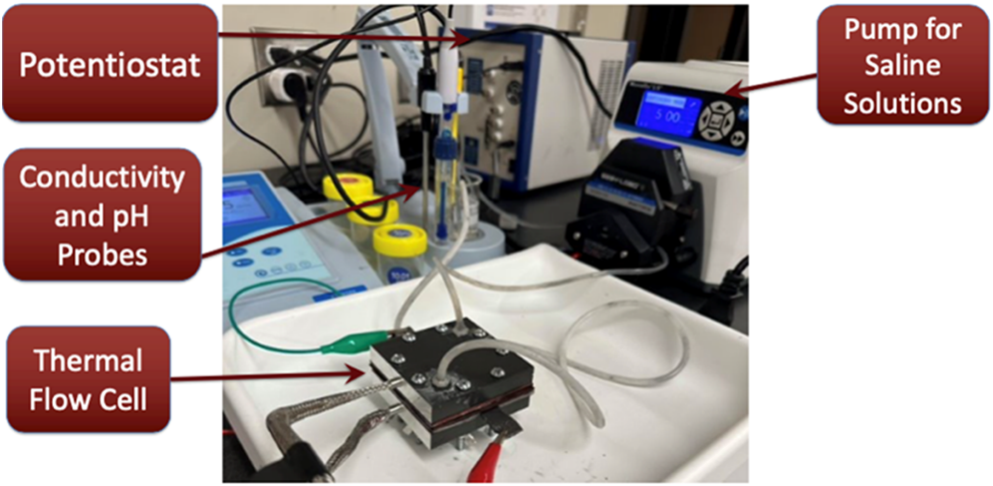
Experimental setup for the CDI cell.

Once the electrode has been fully prepped, the flow cell (Figure 1) can be constructed and used for desalination experiments. The flow cell consists of two metal plates made of anodized aluminum (1/2 in., Multipurpose 6061, McMaster-Carr), three rubber gaskets (high-temperature silicone rubber, McMaster-Carr, 1 mm) used for spacing and separation, and two graphite conducting plates. The electrode on the graphite foil (43078, 0.13 mm, Alfa Aesar) will be cut out into a diamond shape which will be placed on the conducting sheets, covered with a Kimwipe, and sprayed with deionized water, and then placed to face toward each other.

The metal plates have a hole on the top of each plate that is used for the insertion of a conducting rod to be inserted. The end plates were made of anodized aluminum (Multipurpose 6061, McMaster-Carr). The conducting rods used as heating elements were made of stainless steel with internal K-type thermocouple sensors embedded to determine steady-state temperature for an applied current (McMaster-Carr). The rods were 1/4 in. in diameter and 2.5 in in length, a size that fit well into the end plates of the cell. These rods are connected to a power source and applied with a specific current value to generate a certain temperature for the flow cell. To ensure proper heating and consistent temperatures, a calibration plot was made for both the left and right channels of the power source connected to the conducting rods (Figure S1A). The two channels have not been used equally in previous experiments, so this causes one rod to need more current to induce the higher temperature needed.

In the experiments, conductivity values are recorded during various charging and discharging cycles. The overall goal is to be able to relate this value to see the change in the concentration of sodium chloride. Using points at various known concentrations, a second calibration plot (Figure S1B) was constructed to relate the measured conductivity value to the known concentrations. From this, the average removal of NaCl concentrations for the different cycles of the CDI experiment could then be determined.

The full experimental setup will involve the complete flow cell with conducting rods, saline solution, Masterflex pump and tubing, pH and conductivity probes, potentiostat, and a conductivity probe connection port. The chemicals used for electrode construction are from Sigma-Aldrich.

The same flow cell in Figure 1 will be used for each experiment while varying the concentration of the saline solutions and the temperature of the testing environment. The concentrations of NaCl used are 2, 5, 10, 20, and 40 mM, and the temperatures used are 20 (room temperature), 30, 40, and 50 °C. Each combination of these parameters will be a separate test for the CDI experiment, and for each test, the conductivity values will be recorded.

## Theory

### Computational CDI Development

The CDI cell is considered with a monovalent salt (in this case, NaCl, such that valencies z^–^ and z^+^ are assumed as unity). The carbon electrode consists of two primary regions: macroporous and microporous. Generally, the microporous region is above 50 nm and the microporous region is reported to be in the range of 4 nm or below.^18^ The pore size affects the electrostatic potential of the electrode. Smaller pores are favored when removal is higher since this enhances the surface area through which ions can be adsorbed. The challenge with micropores is in the overlap of the double layer, which complicates the removal of lower concentrations. It is said that a healthy distribution between the two should exist, with average pore size in the range of macropores, to reap the benefits of both the macro and micropores.^44,45^

A charge balance within the electrode consists of three different types of charge: ionic, electronic, and chemical charge:
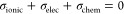
1

Thus, if σ_chem_ = 0, as in traditional CDI, the magnitudes of charges are equal and they are commonly assigned a singular value. The charges are volume-based, averaged by the micropore volume. If divided by the Faradaic constant, then the charges will be in units of mol/m^3^, or mM, the same unit as ion concentration *C*. All types of charge values will henceforth be referred to as units of molar concentration.

**Figure 2:**
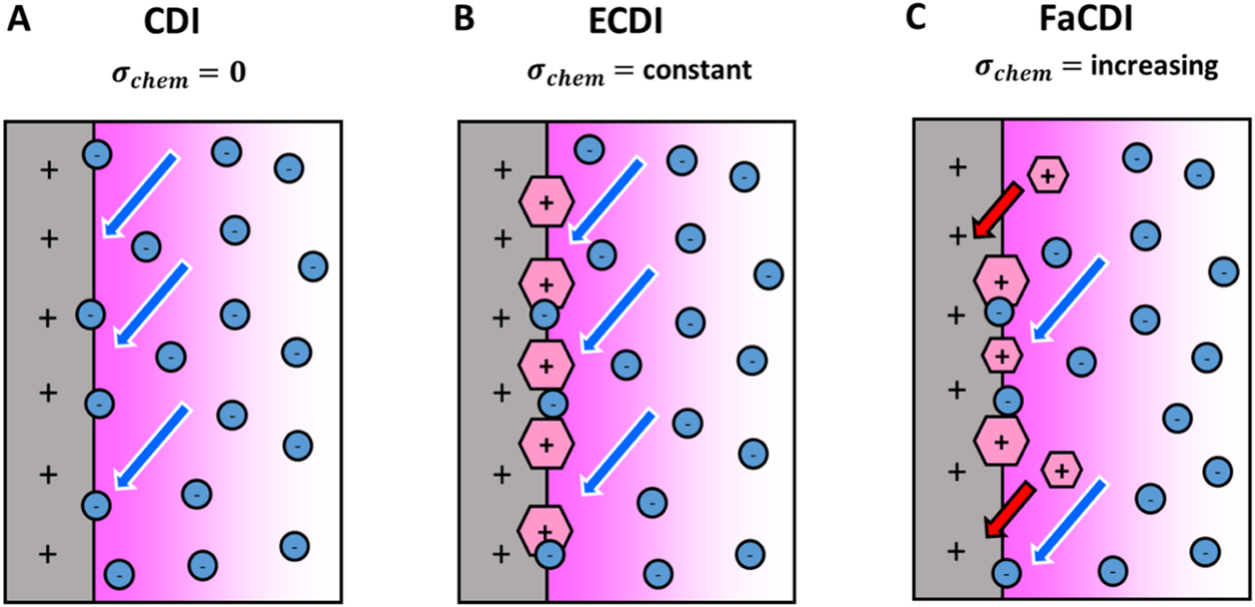
Schematic of the charging of micropores of a CDI cell, depicting (A) traditional CDI process with no chemical charge, (B) enhanced CDI process with fixed/immobile chemical charge, and (C) Faradaic CDI process with variable chemical charges, which increase during the charging stage and decrease during discharging.

Three different cases were considered for CDI testing: traditional CDI (Figure 2A), in which no chemical charge is used (σ_chem_ = 0), enhanced CDI (ECDI) (Figure 2B), in which a chemical charge value σ_chem_ is fixed to alter the point of zero charge (PZC) of the system at equilibrium, and Faradaic CDI (FaCDI) (Figure 2C), in which chemical σ_chem_ varies based on redox reactions taking place at the electrode surface.

As this work focuses primarily on how the governing behavior for these electrodes affects overall cell performance, the spatial variations within the electrode structure and the feed will be neglected. This is an important simplification to make and is reasonable when assuming mass transfer limitations are negligible and the fluid is well mixed within the cell. With the mD model, assuming a symmetric cell, we then can determine the total cell voltage by
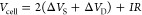
2

where *V*_S_ represents the potential in the Stern layer and *V*_D_ is the potential in the Donnan layer. The cell voltage also comprises the ohmic drop *IR*, in which the resistance varies as a function of cell concentration, as well as geometry and electrode/electrolyte properties. Higher temperatures will generally increase conductivity in the electrolyte, which will decrease ohmic resistance.^46^ This will result in larger ohmic drops at larger temperatures, but generally, this is only apparent when the salt removal capabilities are maximized.

The Stern layer voltage varies with the electronic charge:
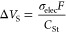
3

where *C*_St_ represents the Stern layer capacitance, given in units of F/m^3^. This capacitance is fit to a parabolic profile:^47^
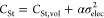
4

Here, *C*_St,vol_ = 150 F/m^3^ and α = 30 F m^3^/mol^2^ will be used. The differences between eq 4 and constant Stern layer capacitance are negligible but can still exist. The Stern layer capacitance has also been reported to display some variations with the operating temperature, but the exact variation has been somewhat unclear in the literature.^48−50^ Since temperature variations are not as large in this study, this contribution will be neglected.

For the diffuse layer, the mD model assumes that the micropore diameter is much smaller than the Debye length λ_D_, and as such, the Debye length is not explicitly needed in the calculation of the diffuse layer potential. Instead of a Debye length, the mD model assumes that there exists a Donnan potential located at the macropore–micropore interface.

This Donnan potential is given as^47^
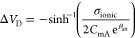
5

where μ_att_ is an ionic-based attraction term between charges in the porous carbon structure. This is usually much more significant for examining CDI at higher concentration values and has been used for enabling mD to function for both high and low ranges for CDI. However, since only lower concentration values for CDI have been examined, the value and contribution of μ_att_ will be neglected in this study.

The primary difference between CDI, ECDI, and FaCDI is the presence of the chemical charge σ_chem_, which alters the performance of the double layer as given by eqs 3–5. The micropore concentration is related to the macropore concentration using the Boltzmann distribution:
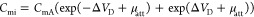
6

where again the attractive term μ_att_ is neglected in this study. If the cell is assumed symmetric, then the redox-active polymer undergoes a single electron exchange during the charging process: e.g., O + e^–^ → R^–^ for oxidant O and reductant R. For the Faradaic reactions, the chemical charge σ_chem_ varies as a function of the Stern layer voltage C_St_ and other properties constant based on the characteristics of the electrode.

The equilibrium (dimensionless) Stern layer voltage Δ*V*_S,eq_^0^ is given as
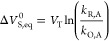
7

For the transient analysis, the setup of ref (27) is followed similarly, but make a few new additions. The mass balance over the cell is given as
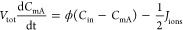
8

where the ionic fluxes are assumed equivalent for the anode and the cathode, here approximated as
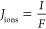
9

For the Faradaic reactions in FaCDI, chemical charges change over time according to
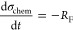
10

The chemical charge generation follows first-order kinetics with reaction rate *R* given as
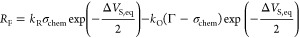
11

where Γ represents the maximum chemical charge that may exist at the electrode surface (a constant) and Δ*V*_S,eq_ is the Stern layer voltage. The current within the cell, *I*, is related to the ionic charge by
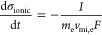
12

where *m*_e_ and *v*_mi,e_ represent the mass and gravimetric micropore volume of the electrode. Finally, the electrical charge magnitude at the surface can be related to chemical and ionic charges using the electroneutrality condition, as shown by eq 1. When considering batch mode tests, the most important modification to make is to the mass balance (eq 8). In batch mode, the inlet concentration is not constant, but will change gradually as a function of the total batch volume, the outlet feed concentration, and the total volume that passes through the cell at an instant in time. As such, the updated inlet concentration is updated as
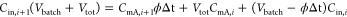
13where Δ*t* represents the change during one instant in time.

Unless where explicitly stated, all constant parameters, material, and operational parameters were borrowed and replicated from the previous studies^19,27^ and examined to fit the new evaluation and cycle developments.

In CDI cycles, an important parameter to assess and compare overall performance is the thermodynamic energy efficiency (TEE), given as the ratio of the minimum possible required energy and input cycle work:
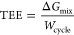
14

The cycle work can be evaluated by integrating the voltage over the changes in charge over the entire cycle.^51,52^ The minimum required input energy is called the Gibbs energy of mixing, Δ*G*_mix_, and is given by^53^
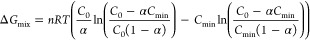
15where *n* represents the van’t Hoff factor, which for 1:1 electrolytes assumes a value of 2. α represents the water recovery ratio or the portion of the salt water volume that is used for salt removal. *C*_0_ represents the starting concentration in the stream and *C*_min_ is the diluate or minimum concentration, also referred to using *C*_D_. Therefore, when considering the relevant limit, this mixing energy is maximized when *C*_min_ is lowest and α is highest.

### Adsorption Thermodynamics

Most adsorption isotherms studied have looked at gas adsorption, but they can also be used for liquid solutions, varying as a function of concentration. Adsorption isotherms have been modeled in several desalination studies.^54−58^ Of the models that exist to predict the variation of electrosorption as a function of equilibrium concentration, the two most cited in the literature are the Langmuir isotherm and the Freundlich isotherm.

The Langmuir isotherm model, while initially developed for gas–solid adsorbent pairs,^59^ is also applicable to liquid solutions. In such solutions, the adsorption varies as a function of concentration at equilibrium, *C*_eq_, typically given in milligrams per liter. The Langmuir model assumes fully homogeneous, monolayer adsorption with no interaction of the adsorbed species in the plane of the surface. Furthermore, the model assumes that there is a limit to the maximum quantity of total adsorption by the adsorbate. The governing equation for the Langmuir adsorption isotherm is given as
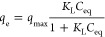
16

where *K*_L_ is the Langmuir constant, given in units of *q*_max_, which represents this maximum adsorption. The concentration of *C*_eq_ is determined as the equilibrium concentration for the adsorption process. In continuous mode processes this can be assumed as the inlet concentration, but if the process is done in batch mode with a finite volume constantly circulated during adsorption, the equilibrium concentration is given as the average between inlet and dilute concentration.

The Freundlich isotherm extends beyond monolayer adsorptions of the Langmuir model and assumes that multiple layers exist through which the sorbent can be stored within the sorbate. Unlike the Langmuir model, however, the Freundlich isotherm is semiempirical^60^ and does not have a purely theoretical basis behind its values. The Freundlich isotherm is preferred when lower concentrations of solute are used,^61^ and commonly for desalination it is directly compared with the Langmuir adsorption process. The Freundlich isotherm is given as a power relation:
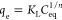
17

where *K*_F_ is a constant, related to the heat of adsorption, given in units of (L/mg)1/*n*, and 1/*n* is a constant power parameter, which indicates the heterogeneity of the solution and indicates the tendency for which the sorbent readily adsorbs.

For desalination processes, adsorption isotherms can be developed by examining salt removal with experimental operating conditions as the control and initial feed concentration as the variable. A plot of *C*_eq_ vs *q*_eq_ can be fitted to the Langmuir and/or Freundlich adsorption isotherms to predict the extent to which monolayer or multilayer adsorption holds under the given conditions based on *R*^2^ values.^61^ The chosen equilibrium constant *K*_eq_ is then multiplied by appropriately related parameters to make its value dimensionless. Regardless of which isotherm is used, it is important to perform this unit conversion correctly, as it has been cited as a source of error in various adsorption isotherm literature.^62^

If evaluating a range of different temperatures as well as concentrations, isotherms can further identify further characteristics of the adsorption process. The equilibrium constant *K*_eq_ can be related to Gibbs free energy of adsorption (here referred to as Δ*G*) by^63^
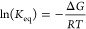
18

when Δ*G* < 0, the process is said to be spontaneous; positive values are considered nonspontaneous. The Gibbs energy can also be used to calculate related enthalpies and entropies of adsorption:
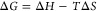
19

The relationship between Δ*G*, Δ*H*, and Δ*S* can be plotted as a linear variation with *T*. More specifically, the variation of ln(*K*_eq_) vs 1/*T* can be used to determine Δ*H* and Δ*S* via linear regression. Thus, Δ*H* and Δ*S* can be used to further characterize the adsorption process. If Δ*H* is positive, it is indicative of an endothermic reaction; if it is negative, it is indicative of an exothermic reaction. Furthermore, the magnitude of the enthalpy of adsorption allows for the prediction of characteristics for some adsorption processes: If the enthalpy values are below 84 kJ/mol, the adsorption is considered physiosorption; whereas values from approximately 80–420 kJ/mol are considered chemisorption.^59^ Alternatively, it has been suggested that chemisorption process can occur if the Δ*H* values are negative, regardless of their magnitude.^64^ The entropy of adsorption indicates the affinity between the sorbent and the sorbate. If the value is higher, then there is more favorable adsorption due to greater degrees of freedom from the sorbate. If the entropy value is negative, the activity of the sorbent at the solid–liquid interface is reduced, essentially making the adsorption process more difficult.^28^

The determination of Δ*H* and Δ*S* experimentally has been done for salt water desalination on various electrodes, for the NaCl sorbent as well as other ions in aqueous solutions.^64,65^ However, the breadth of experimental data collected is limited, since ranges of both concentrations and temperatures are required to determine a single Δ*H* and Δ*S* value. As a result, the viability of exploring fundamental adsorption processes with different values/ranges of multiple parameters is further complicated. Computational studies can relieve the arduous task of running many experiments and rapidly assess the sensitivity of Δ*H*, Δ*G*, and Δ*S* to a wide range of operating conditions.

To illustrate clearly to the reader, a full example set of thermodynamic isotherms for all three mechanisms, along with associated fitting values, is included in the Supporting Information (Figure S2 and Tables S2–S4). As the results shown for this case had not yet been fit to experiments, the primary figures in this paper will focus on reflecting and comparing to the experimental data that was obtained.

## Results and Discussion

### Experimental CDI Studies

The electrode salt removal is first tested with the same electrode using different concentrations of NaCl and variable testing temperatures applied by the conducting rods and the metal plates of the flow cell. The concentrations of sodium chloride used are 2, 5, 10, 20, and 40 mM and the temperatures used for the tests are room temperature of about 20, 30, 40, and 50 °C. Each concentration will be tested at each temperature, and the conductivity values will be recorded. The cells are charged (at 1.2 V) and then discharged (at −1.2 V) over a total of five cycles, with each charging and discharging stage lasting 2 h. To make the results easier for the reader to interpret, the plots of concentration in each case are normalized relative to the starting concentration for each test run. Only two cycles are shown out of the five runs for purposes of clarity and to demonstrate stability in the cycles.

**Figure 3:**
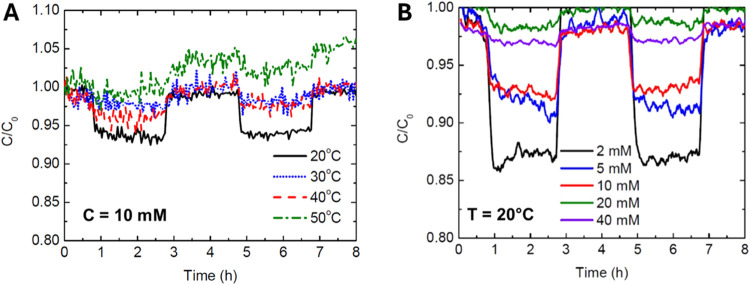
Plot of relative concentration salt removal for CDI experimental studies. (A) Dependence on temperature. (B) Dependence on the starting concentration.

When different temperatures are evaluated (Figure 3A), the challenges associated with removing small amounts of salt at higher temperatures are revealed. At room temperature (20 °C), the CDI cell can remove up to 6–7% of the salt consistently and return the temperature to approximately the original value. With the intermediate temperatures, this removal becomes more difficult, as only a smaller amount can be removed, and the removal becomes harder with each subsequent cycle. Finally, at the maximum temperature of 50 °C, the cell cannot effectively remove salt and the concentration experiences a net increase during the discharge stage. This is seen in cases of oversaturation and indicates that additional ions may be entering the system under these conditions. Furthermore, while it has been noted that ionic conductivity increases with temperature, there were no major fluctuations and temperature had stayed at nearly the set-point values. From the temperature-based results, maximum salt removal at each temperature can be obtained for a given concentration and used to generate the thermodynamic adsorption parameters.

The removal can also be examined by using different salt concentrations. To do this, we will view an isotherm with each concentration value and observe how the relative concentration is removed for each temperature. The isotherms will use the concentration removal values from the tests and fit these values with the models listed in eq 16 to test for the adsorption by the cell. Figure 3B shows the removal of salt relative to the starting concentration values for all of the solution concentrations used at the same temperature.

**Figure 4:**
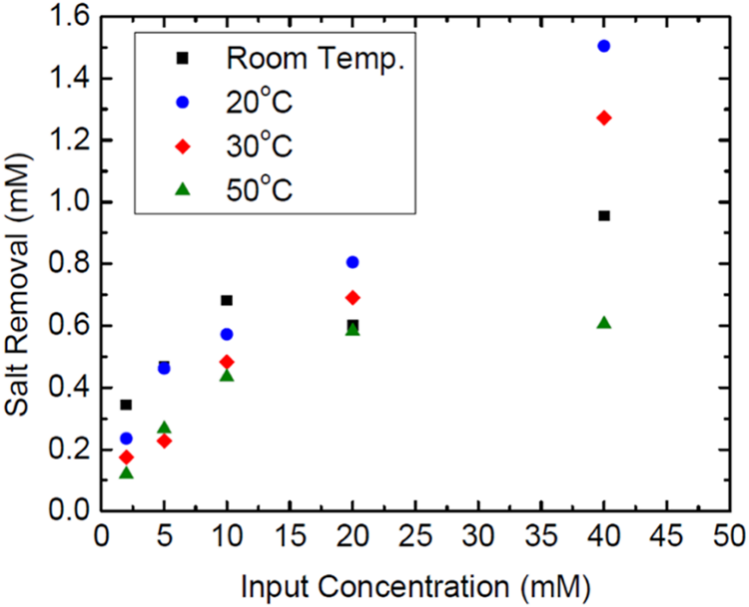
Maximum removal per cycle vs concentration at varying temperatures.

At different temperatures, the maximum salt removal is then compared as a function of the starting concentration. While previous graphs suggested that removing the salt becomes more difficult at higher concentrations, the amount of salt removed at higher concentrations is increased. The results are taken as the maximum possible for all cycles and not the average or last cycle to best determine the cell’s maximum removal capabilities. At higher concentrations, larger deviations are apparent (Figure 4).

At maximum salt removal, temperature variations are not consistent, although the decrease in salt removal with temperature is pronounced when the starting concentration is lower. Cyclic voltammetry tests run prior to the long-term experiments suggested that higher temperatures could benefit short-term performance at lower concentrations, but would weaken it at higher concentrations (Figure S3). There, it was shown that peak currents decreased and overall cell capacitance (predicted by the area enclosed) had decreased as well (Figure S4). Nevertheless, capacitance was found to fall within the ranges predicted for CDI electrodes,^66,67^ although some recent studies could obtain considerably higher values.^68,69^

The results from Figure 4 yielded a Δ*H* of −39 kJ/mol, suggesting low potential for physisorption, as well as a high degree of randomness, which would make sense from the data shown. The Δ*S* value obtained was 134 J/(mol K), indicating higher randomness associated with the adsorption.

### Integrating Model with Experiment

The experimental results for the case of room temperature are aligned with a computational model to best fit the modeling parameters used. Table 1 details the key parameters that were used to fit in the model, most of which were constrained based on the experimental parameters used. Here, the cell resistance needed to be maximized to approximately 12,000 Ω for a 1000 m^2^/g electrode area, to obtain a similar salt removal. While the experiment appears to reach an asymptote, it is not as definitively pronounced as the Langmuir model for monolayer adsorption would predict. The Langmuir model also predicts that maximum salt removal is reached quite rapidly, as opposed to the gradual increase as seen by the experimental results. This suggests that maximum removal would ultimately need to be tested for a higher concentration present in the system.

**Table 1:** Key Parameters Used for the CDI Computational Model^a^

parameter	value
cell area	5 cm^2^
electrode mass	180 mg
electrode area	1000 m^2^/g
cell thickness	7 mm
batch volume	40 mL
flow rate	5 mL/min
resistance	12,000 Ω

a Values in this table are rounded, estimated from predicted or averaged experimental values.

**Figure 5:**
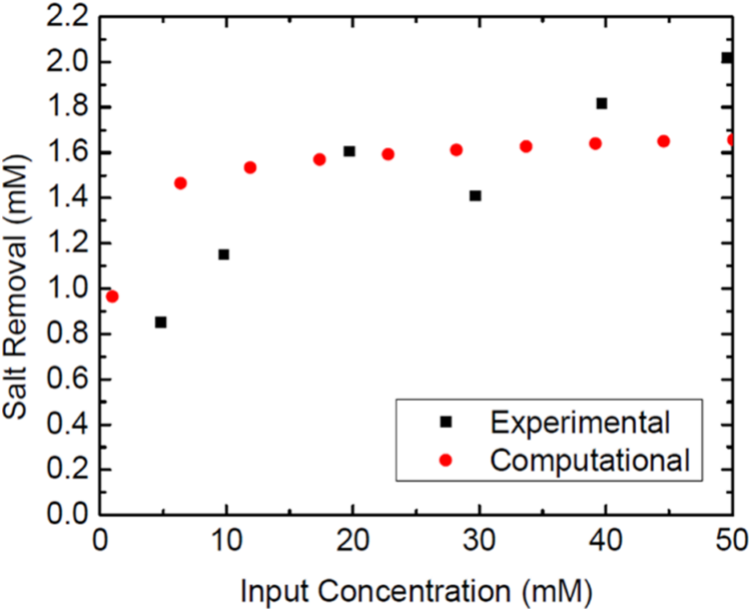
Comparison of experimental and computational results at the base (room temperature) of 20 °C.

At this point, the three different methods of CDI were evaluated and compared against one another (Figure 5). While no experimental data was available for ECDI or FaCDI, this study will consider only computational analysis. The ECDI and FaCDI mechanisms will incorporate the chemical charge term outlined previously, with σ_chem_ = 400 mM for ECDI. For FaCDI, the study used Γ = 800 mM, *k*_R_ = 1.97 s^–1^, and Δ*V*_S,eq_^0^ = 0.1 V.

**Figure 6:**
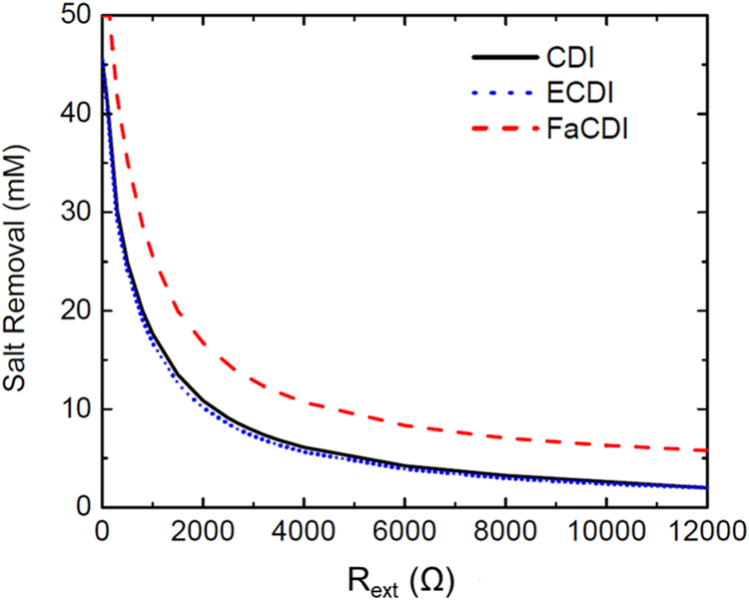
Effect of the external cell resistance on the maximum salt removal capabilities for each of the three CDI adsorption mechanisms studied.

The experimental data served as a starting point for the computational model. To illustrate the performance of the fit, a series of adsorption tests will be used at room temperature and a cell voltage of 1.2 V. Fitting to the experiment necessitated high resistance in the model, although the experimental cell did not adsorb the optimal amount of salt. When cell resistance was decreased, salt removal could quickly be optimized for all three adsorption mechanisms (Figure 6).

The maximum capability for salt removal could be investigated by altering the cell resistance, the only parameter that cannot be determined experimentally. As expected, if the cell resistance were decreased, salt removal could be enhanced. This is similar for CDI and ECDI, but a notable boost of approximately 5 mM is achieved with FaCDI. This increase is consistent with the increase in salt adsorption capacity as outlined in the computational studies outlined in the Supporting Information (Figure S2).

**Figure 7:**
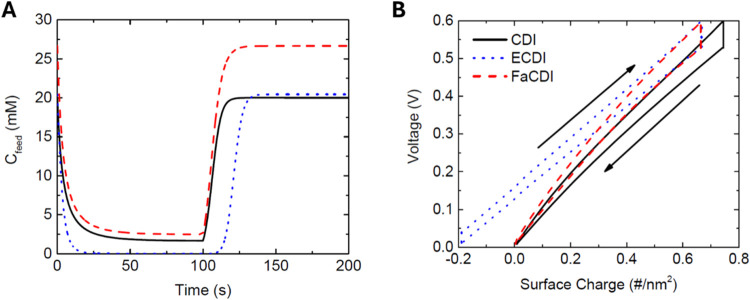
(A) Comparison of the effluent salt concentration for adsorption–desorption cycles in batch mode. (B) Depiction of a four-stage CDI cycle considering variations in cell charge with the applied voltage and concentration. For this cycle, *C*_0_ = 20 mM, *C*_D_ = 1 mM, and water recovery ratio = 0.5.

Short-term tests run in batch mode will determine the feasibility of implementation and consideration of appropriate storage mechanisms. The cycle, when run in batch mode, is shown after charging at 0.6 V and discharging at 0 V for three cycles to ensure that cycling stability is reached. The continuous mode conditions mirror those of ref (27), but performance parameters are further examined here. FaCDI demonstrates a clear benefit in the minimum salt capable of removal (Figure 7A), but comes with the trade-off of higher brine concentrations and higher charging current. FaCDI nonetheless doubled the overall cell charge efficiency; CDI results yielded an overall charge efficiency of 49% while FaCDI approached 98%. ECDI showed an improvement in this case over CDI, reaching a charge efficiency of 65%.

The three mechanisms are also compared in a full CDI cycle incorporating the charging and discharging stages, including two intermediate stages of essentially zero current (constant charge) in which the concentration values are switched. The cycle demonstrated in Figure 7B is for a case of *C*_0_ = 20 mM, *C*_D_ = 1 mM, and water recovery ratio α = 0.5. The cycle operated using four stages: Initial charging from initial concentration *C*_0_ to the diluate concentration *C*_D_, switching to return to *C*_0_ while maintaining constant ionic charge σ_ionic_, discharging to a brine concentration, and switching back to *C*_0_, again at constant σ_ionic_. This operation is thus like the processes described in refs (46,70), and (71).

FaCDI cycles benefit from reduced work through the tuning of the chemical charge during cycling. With ECDI, the voltage range is kept nearly constant, with a surface charge magnitude of 0.05 #/nm^2^. This charge is similar to what had been previously observed,^27^ where ECDI shifted the PZC to approximately 0.5 V. The ECDI cycle reduces the maximum charge reached, it could limit work relative to CDI, but this comes at the expense of ionic charge values dropping below zero as the cycle is further discharged. This negative charge is the result of the fixed chemical charges, which remain active during the discharging stage, when they are not needed, resulting in an increase in unnecessary input work to compensate for the inclusion of these fixed charges. This issue is resolved when the redox active chemical charges are introduced in FaCDI. The chemical charge composition increases gradually during the charging stage to take advantage of the reduced charge during ECDI (oxidation process). During the discharge, the reverse reaction occurs in which the minimum cycle charge returns to the value of nearly zero for CDI. While the voltage ranges remain the same as defined for this steady-state-based cycle, the charge range is decreased by nearly 12%, corresponding to 10% reduction in the network for this particular cycle. The cycle shown in batch mode shows that FaCDI demonstrates a clear benefit in the minimum salt capable of removal, but this also comes with a trade-off of higher brine concentrations and higher current values. Here, a lower voltage is used since electrodes in the model developed were less resistive and thus easier to remove salt.

### Effect of the Applied Voltage

Along with the concentration, the voltage will affect the means of adsorption and related thermodynamic parameters. Experiments with traditional CDI on the in-house cell demonstrated a linear trend with salt removal when voltage was increased over a series of 5 different cycles, for concentrations between 2 and 10 mM (Figure S5). Generally, for CDI, values are kept under 1.2 V, but performance can increase if run using alternative electrode materials and configurations, enabling larger salt removal. In the computational model, the three adsorption methods are then compared to one another under the same set of operating conditions. The goal is to determine the differences in the thermodynamic adsorption parameters.

**Figure 8:**
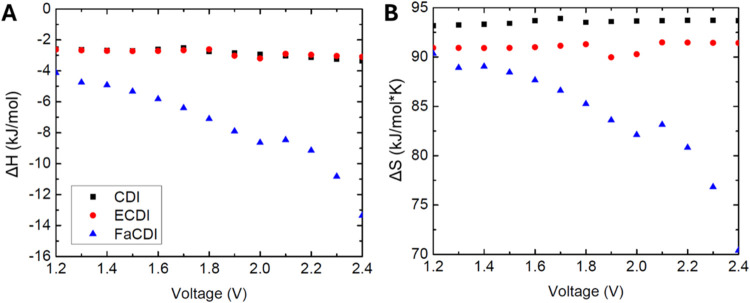
Plot of adsorption values as a function of the input voltage applied to the cell, 1 mM < *C*_feed_ < 20 mM. For low salt removal, this corresponds to an *R*_ext_ value of 10,000 Ω. (A) Enthalpy of adsorption and (B) entropy of adsorption.

When salt removal is low, the adsorption value again does not exceed 0 kJ/mol (Figure 8A). This suggests that, given how the plots are generated, the effect of temperature on removal is reversed. Whereas higher concentrations will see removal decrease with temperature, when salt removal is relatively low, the removal increases with temperature, reversing the trend in the van’t Hoff plot. The temperature dependence is more notable for FaCDI, when enthalpy values become more negative if higher voltage is used. This would further imply that despite energy savings, FaCDI is not optimal when only a small amount of salt is removed, and using traditional CDI will be sufficient. A similar trend can be observed with entropy (Figure 8B), where all values start higher but then decrease at higher applied voltage. This would suggest that greater voltage is more ordered when more salt is removed and that FaCDI is more preferred to use at higher voltages. This coincides with suggestions brought about from previous literature.^72,73^

**Figure 9:**
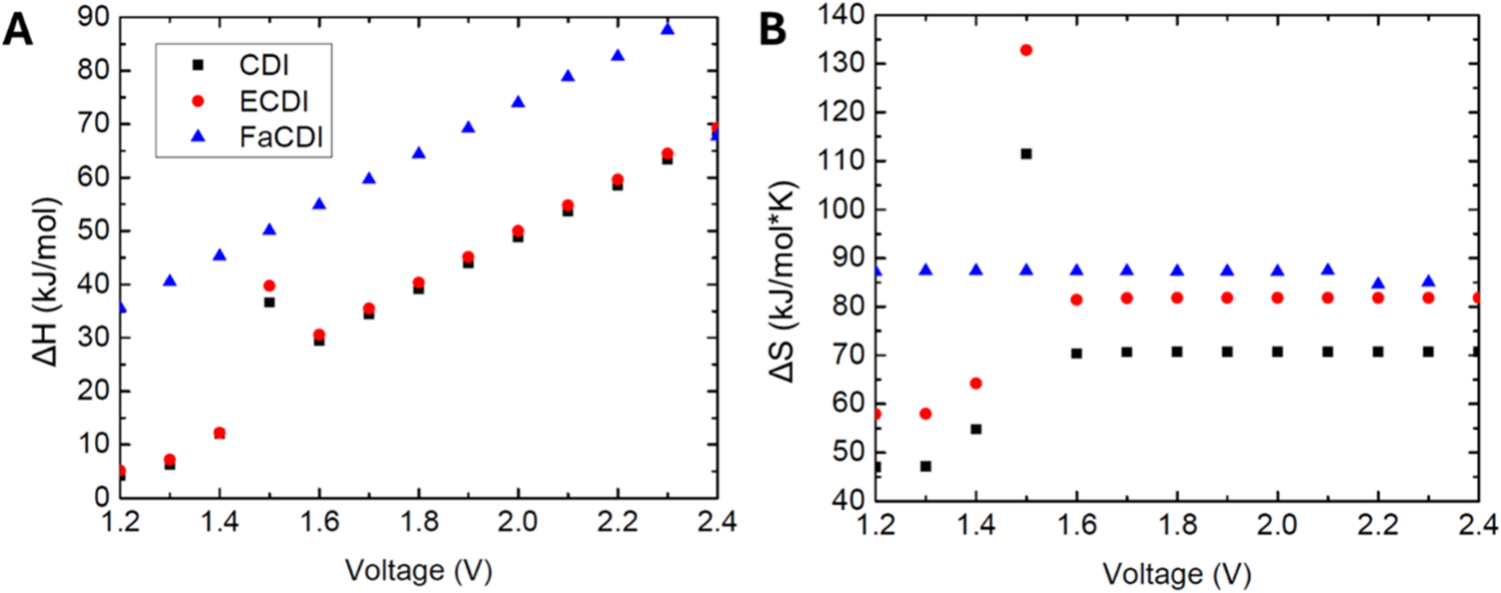
Plot of adsorption values as a function of the input voltage applied to the cell, 1 mM < *C*_feed_ < 20 mM. For low salt removal, this corresponds to an *R*_ext_ value of 100 Ω. (A) Enthalpy of adsorption and (B) entropy of adsorption.

With salt removal increased, notably different trends are seen (Figure 9); when the voltage is increased, all three methods display a nearly linear increase in Δ*H*. It has been shown for all three methods previously that when salt removal is increased, the dependence of performance on temperature follows an inverse relationship; now the Δ*H* values are positive and become larger. The FaCDI is notably higher, and when voltage is maximized, Δ*H* does exceed the generally accepted limit of 80 kJ/mol for a physisorption-based mechanism. It would be expected that should any form of adsorption exceed physisorption and potentially reach the range for chemisorption, it would be the FaCDI mechanism. With Δ*S*, unlike Δ*H*, a maximum appears to be reached at the higher voltages. This maximum is still highest for FaCDI, indicating that the mechanism is still more spontaneous at this removal value, and possesses higher adsorption affinity, despite also having a higher Δ*H* value. The FaCDI maximum of 90 J/mol·K is similar to that shown for lower salt removal. This type of CDI mechanism should generally have some expected ranges for Δ*S*. There is a notable jump at the voltage value of 1.5 V, and this may indicate a challenge in fitting with the isotherm at this value, or a transition in the temperature dependence altering the curves shown in Figure 4. Here, it is expected that this is due to a change in the temperature dependence on salt removal changing from a direct relationship to an inverse one, thus complicating the fits in eqs 18 and 19 and highlighting a limitation in the model at voltages around this value. This does not mean that adsorption characteristics cannot be determined at this value but that temperature dependence needs to be more carefully assessed. Experimental results around this value could help to confirm this if run at different temperatures. For Δ*S*, the values of CDI and ECDI appear to hit a maximum at higher voltages; therefore, this may indicate a maximum/transitional region occurring here.

### Model Sensitivity

One question brought up from the computational study is, how many simulations would need to be run to obtain reliable values for each of these parameters? To do that, cycle time, number of cycles, time step, temperature, and concentration values were all considered. The results operate under the same set of parameters as listed previously, only with a lower external resistance of 500 Ω, to enhance the salt removal. Voltage was kept constant (at 1.2 V), and only the running time for each cycle and the time step used in the simulation were changed.

**Figure 10:**
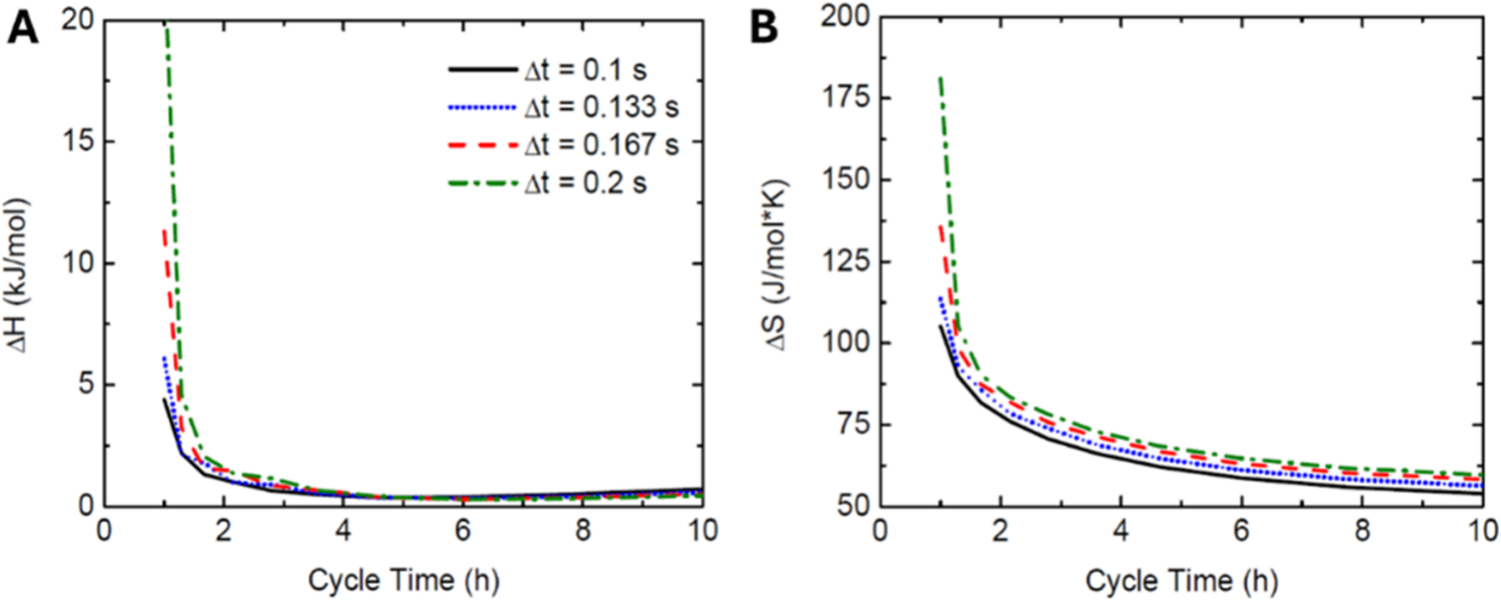
Dependence of the cycle time and numerical time step on Δ*H* and Δ*S* values. (A) Enthalpy of adsorption. (B) Entropy of adsorption.

The results were examined for a CDI case study and suggested a minimum 2 h running time would be required for the enthalpy values to remain stable (Figure 10A). The same graphs also evaluate the dependence on the incremental time step, and results do not appear to change significantly when running time is increased, although smaller times in the range of 0.1–0.2 s would result in substantially larger simulations. Δ*S* values appear to converge slower than Δ*H*, suggesting stability over a longer running time in the system coincides with more organization as the Δ*S* value is decreased (Figure 10B). The number of cycles was also tested in a separate study, but negligible changes in either value were noticed. Therefore, it is concluded that among three consistent cycles in each study result in convergence of the Δ*H* and Δ*S* values converging. While this is sufficient in the model, it also will need to be confirmed experimentally.

**Figure 11:**
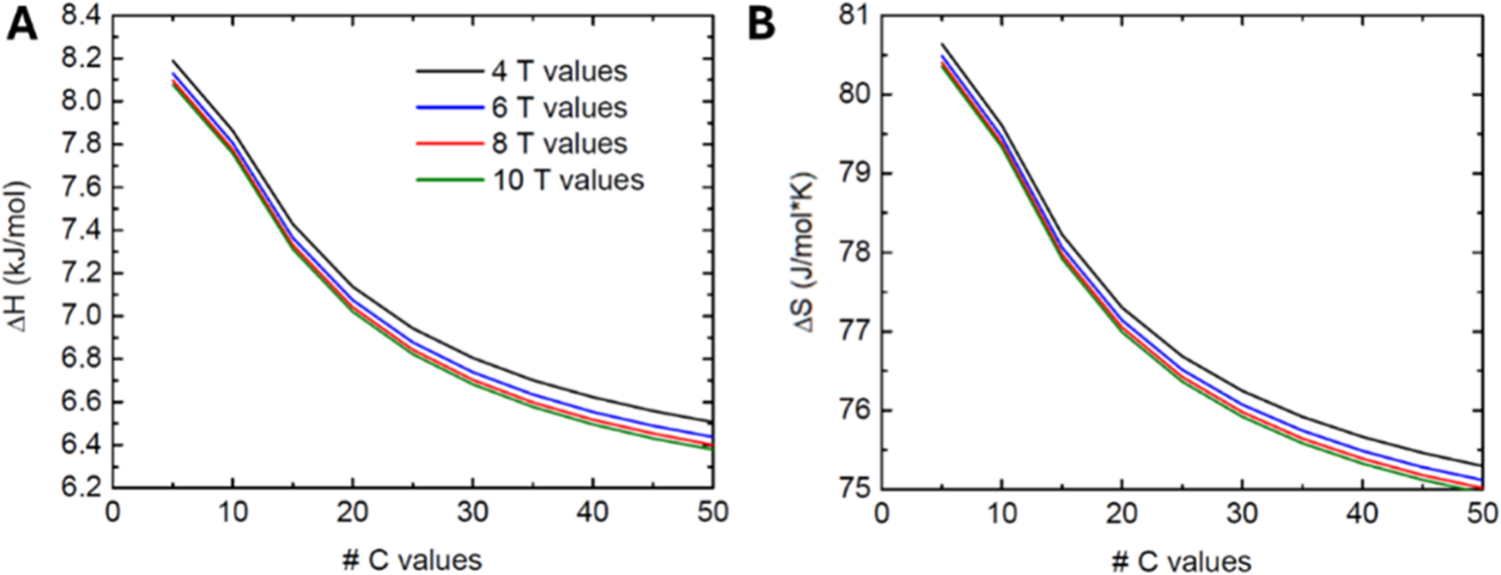
Dependence on the concentration and temperature values used for isotherm fit to generate Δ*H* and Δ*S* values. (A) Enthalpy of adsorption. (B) Entropy of adsorption.

Another interesting result is when the number of data points selected for concentration and temperature was changed (Figure 11). The ranges of concentration and temperature were kept the same in each study. The results immediately reveal that increasing the number of concentration values is much more valuable and suggest that more concentration values should be prioritized over more temperatures.

Additionally, there is a need to look at the number of temperature and concentration points needed for a higher accuracy. This could be done by starting with the baseline values of the temperature and concentration values and then altering them to see the effect on the changes in the adsorption parameters. Theoretically, the number of values selected should not change the parameters, as the adsorption isotherms should be continuous. However, the number of values used could change the accuracy of the fitting parameters.

Figure 11 also shows that more temperature values would not alter adsorption parameters as significantly, but increasing the number of concentration values has a greater effect. This may be due to the influence of all of the temperature values on each unique *K*_L_ and *q*_m_, all of which inherently affect the isotherm fit to the concentration. The results also suggest that even more values could be used to establish true convergence, but running this many individual experiments alongside temperature values would be unrealistic. In summary, while four temperature values (as had been shown in previous studies as well) should be sufficient, future experiments should aim to increase the number of concentration values, to whatever extent may be reasonable.

## Conclusions

This work develops a methodology for predicting thermodynamic adsorption parameters for various CDI techniques and uses an experimental setup to set a baseline for initial parameters used in the model. The experimental setup focuses on traditional CDI at various concentrations and temperatures to determine the extent of maximum salt removal under a small CDI cell configuration. As running many experiments is cumbersome, the computational model serves as a means through which thermodynamic adsorption parameters can be rapidly assessed. The value in this approach comes from integrating the theory for the micropore-based modified Donnan model with experimental results, providing some guidance on predicting the adsorption parameters, which reveal the performance of the electrode and can suggest means for improvement to enhance adsorption.

Experimental data from a small CDI cell was used as an initial means of verifying the adsorption characteristics of the model, and selected parameters from the model were adapted to best fit these experimental data. The model was then extended to encompass alternative modes of CDI, ECDI, and FaCDI, with fixed and variable chemical charges. The computational results showed that FaCDI could maximize salt removal, operate in cycles with higher efficiency values and greater energy savings, and have the highest adsorption values when salt removal was maximized. Increasing the voltage would also increase the magnitude of values of both parameters but would reach a maximum value for Δ*S* of up to 90 J/mol·K, while Δ*H* would continue to increase. From the Δ*H* values, all adsorption mechanisms covered in this study under different operating conditions displayed physisorption, although FaCDI notably began to transition into the chemisorption range at the highest voltage, which should be expected. Additional experiments should be run with additional adsorption parameters to select better operating parameters and ensure consistency between computational and experimental results.

## Supporting Information Available

Parameters for electrode preparation (heating temperature and time), calibrations for conductivity and temperature in the CDI cell, example isotherms for all adsorption mechanisms and corresponding fitting values, cyclic voltammetry tests to demonstrate and compare the cell’s capacitance as a function of temperature and concentration, and influence of applied charging voltage on salt removal as a function of concentration at room temperature (PDF)
