# Supplementary material for: Thermodynamic Evaluation of Electrode Storage for Capacitive Deionization
Source: ACS Omega. 2025 Mar 4;10(10):10139–51. doi: 10.1021/acsomega.4c08707 (PMC11923673; doi:10.1021/acsomega.4c08707)
Supplement: Supplementary file 1 — ao4c08707_si_001.pdf [file ao4c08707_si_001.pdf]

Supporting Information:

Thermodynamic Evaluation of Electrode Storage for Capacitive Deionization

Daniel Moreno * [1], Hunter Nelson [1], Grant Cary [1], Devon Parker [1], Pablo Skaggs [1]

1. Missouri State University, Springfield, MO, USA

* Corresponding Author, danielmoreno@missouristate.edu

Table SI1. Electrode construction parameters with accompanying conductivity difference values, correlating to salt removal.

Temperature (°C)	Duration of Heating (hr)	Conductivity Diff. (µS/cm)
**150**	4	102
**150**	3	119
**150**	2	113
**150**	1	99
**135**	4	102
**120**	4	95

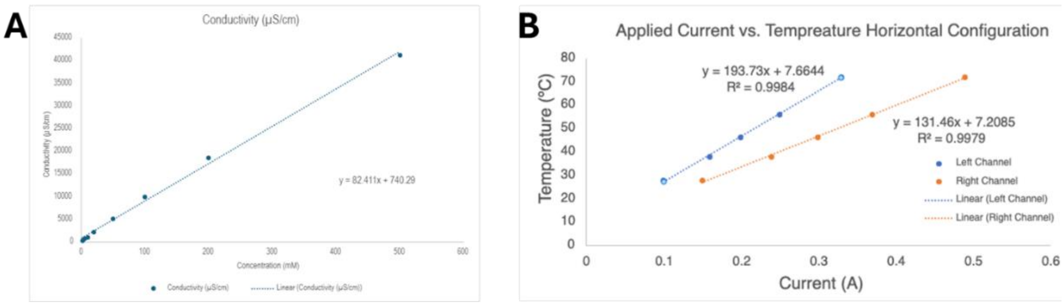

Figure SI1. (A) Conductivity vs. concentration calibration plot. (B) Applied current vs. temperature calibration plot.

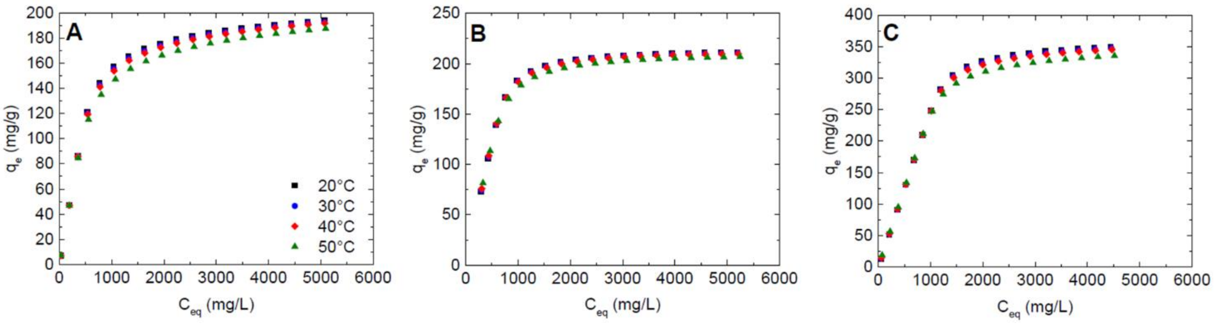

Figure SI2. Batch-mode Langmuir adsorption isotherms. (A) CDI, (B) ECDI, (C) FaCDI.

Table SI2. Fitting parameters for Langmuir/Freundlich isotherms and regression coefficients of CDI, short-term.

Parameter	20°C	30°C	40°C	50°C
K_L	0.00149	0.00153	0.00157	0.00171
K_F	0.2805	0.2759	0.2716	0.2562
q_m	265.71	262.57	259.56	248.73
n	0.3240	0.3208	0.3178	0.3079
R^2 (Langmuir)	0.9289	0.9295	0.9304	0.9361
R^2 (Freundlich)	0.7573	0.7602	0.7633	0.7781

Table SI3. Fitting parameters for Langmuir/Freundlich isotherms and regression coefficients of ECDI, short-term.

Parameter	20°C	30°C	40°C	50°C
K_L	8.4406E-4	8.5862E-4	8.7336E-4	9.3366E-4
K_F	0.5472	0.5463	0.54551	0.5429
q_m	303.13	298.06	293.08	274.10
n	1.0999	1.1025	1.1058	1.1251
R^2 (Langmuir)	0.9981	0.9981	0.9982	0.9984
R^2 (Freundlich)	0.8557	0.8575	0.8594	0.8673

Table SI4. Fitting parameters for Langmuir/Freundlich isotherms and regression coefficients of FaCDI, short-term.

Parameter	20°C	30°C	40°C	50°C
K_L	2.4801E-4	2.5649E-4	2.6542E-4	3.0577E-4
K_F	0.7023	0.6948	0.6871	0.6554
q_m	1021.4367	989.7618	958.6854	841.8838
n	3.0896	2.6861	2.3700	1.5955
R^2 (Langmuir)	0.9982	0.9979	0.9976	0.9961
R^2 (Freundlich)	0.9113	0.9094	0.9075	0.9002

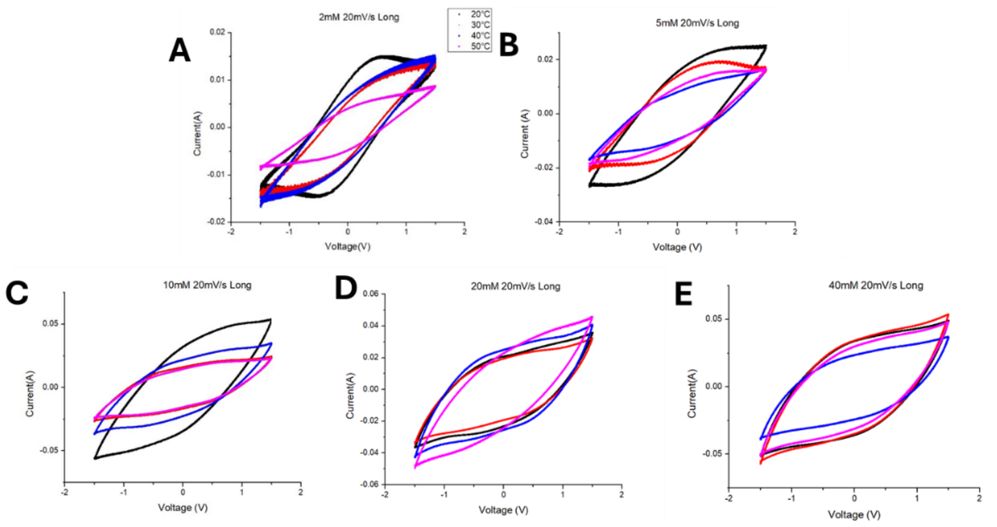

Figure SI3. Cyclic voltammetry plots for different concentrations used. (A) 2 mM, (B) 5 mM, (C) 10 mM, (D) 20 mM, (E) 40 mM.

Electrode capacitance was calculated from the CV curves in Figure SI3 using the following equation:

$$C = \frac{\int IV\, dV}{2mv\Delta V}$$

where I and V represent the current and voltage during the CV test (shown in Figure SI3), m represents the mass of the electrode (here, taken as 180 mg or 0.18 g), ν is the scan rate (20 mV/s), and ΔV is the voltage range (here, 3 V as illustrated in Figure SI3). This value can be computed for each curve in Figure SI3, resulting in a total of 20 calculated capacitances (4 temperatures and 5 concentrations).

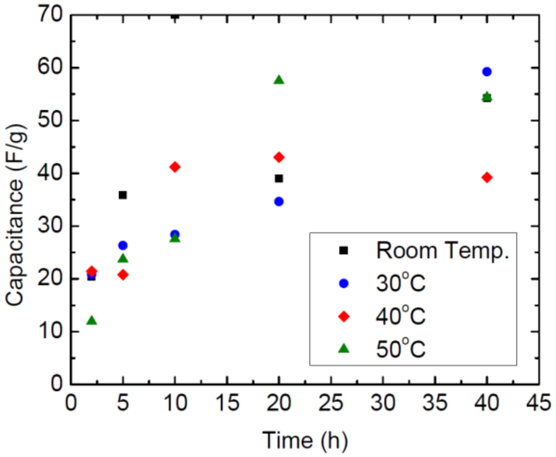

Figure SI4. Calculated capacitance values as a function of concentration and temperature.

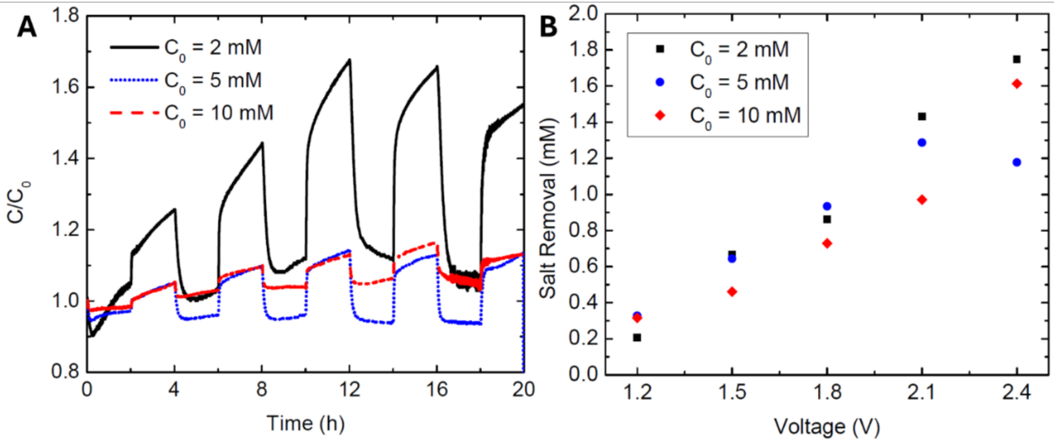

Figure SI5. (A) Salt removal for experimental CDI tests relative to the starting concentration C_0, with voltage increasing linearly between 1.2 V and 2.4 V with each successive cycle. (B) Maximum salt removal capabilities as a function of starting concentration for each successive charging voltage. The discharging voltage remained constant at -1.2 V.
